# Hepatitis B Co-Infection is Associated with Poorer Survival of HIV-Infected Patients on Highly Active Antiretroviral Therapy in West Africa

**DOI:** 10.4172/2155-6113.S3-006

**Published:** 2013-06-29

**Authors:** Nimzing G Ladep, Oche O Agbaji, Patricia A Agaba, Auwal Muazu, Placid Ugoagwu, Godwin Imade, Graham S Cooke, Livia Vivas, Sheena Mc Cormack, Simon D Taylor-Robinson, John Idoko, Phyllis Kanki

**Affiliations:** 1 Hepatology Unit, Department of Medicine, Imperial College London, St Mary’s Hospital Campus, South Wharf Road, London W2 1NY, United Kingdom; 2 AIDS Prevention Initiative in Nigeria & Jos University Teaching Hospital, 2 Murtala Mohammed Way, PMB 2076, Jos, Plateau State, Nigeria; 3 Department of Family Medicine, University of Jos, Plateau State, Nigeria; 4 Medical Research Council Clinical Trials Unit, Aviation House, 125 Kingsway, London, WC2B 6NH, United Kingdom; 5 National Agency for the Control of AIDS, Plot 823, Ralph Sodeinde Street, CBD, Abuja, Nigeria; 6 HarvardSchool of Public Health, 677 Huntington Avenue, Boston, MA 02115, USA

**Keywords:** Mortality, Hepatitis B surface antigen, HIV, CD4, HAART, Survival, Africa

## Abstract

**Background:**

Hepatitis B has been reported to be high in HIV-infected African populations. However, the impact of this co-infection on the survival of HIV-infected Africans on long-term highly active antiretroviral therapy (HAART) remains poorly characterised. We investigated the impact of HBV/HIV co-infection on survival of HIV infected patients undergoing antiretroviral therapy in a West African population.

**Methods:**

This was a clinic-based cohort study of HIV-infected adults enrolled in Nigeria, West Africa. Study subjects (9,758) were screened for hepatitis B and hepatitis C at HAART initiation. Kaplan-Meier survival and Cox proportional hazards models were used to estimate probability of survival and to identify predictors of mortality respectively, based on hepatitis B surface antigen status. All patients had signed an informed written consent before enrolment into the study; and we additionally obtained permission for secondary use of data from the Harvard institutional review board.

**Results:**

Patients were followed up for a median of 41 months (interquartile range: 30–62 months) during which, 181 (1.9%) patients died. Most of the deaths; 143 (79.0%) occurred prior to availability of Tenofovir. Among those that were on antiretroviral therapy, hepatitis B co-infected patients experienced a significantly lower survival than HIV mono-infected patients at 74 months of follow up (94% vs. 97%; p=0.0097). Generally, hepatitis B co-infection: HBsAg-positive/HIV-positive (Hazards Rate [HR]; 1.5: 95% CI 1.09–2.11), co-morbid tuberculosis (HR; 2.2: 95% CI 1.57–2.96) and male gender (HR; 1.5: 95% CI 1.08–2.00) were significantly predictive of mortality. Categorising the patients based on use of Tenofovir, HBV infection failed to become a predictor of mortality among those on Tenofovir-containing HAART.

**Conclusions:**

HBsAg-positive status was associated with reduced survival and was an independent predictor of mortality in this African HIV cohort on HAART. However, Tenofovir annulled the impact of HBV on mortality of HIV patients in the present study cohort.

## Introduction

Viral hepatitis infection is the leading cause of cirrhosis and primary liver cancer in Africa [1], where HBsAg prevalence is up to 20% in the general population [2]. Owing to shared routes of transmission, hepatitis infection rates among HIV-infected patients are significantly higher than in the general population [3,4].

Since the global scale-up of HAART, numerous studies have shown that the efficacy of HAART in suppression of HIV in Africa is comparable to that obtained in resource-rich countries [5–7], though there are conflicting data on whether HBV co-infection affects HIV suppression [8–10]. In the developed world setting, liver disease has emerged as a leading cause of death in the era of HAART [11–13] and viral hepatitis co-infection plays an important role in the progression to cirrhosis in hepatitis B and hepatitis C patients, who are co-infected with HIV [14]. This pattern has continued despite increasing availability and administration of HBV-active HAART.

The role of viral hepatitis and consequent chronic liver disease on mortality has not been widely studied in HIV-infected African populations [15,16], but some studies have noted high death rates during the initial period of ART [17,18]. However, most of the published studies have been small [5–7]. We have recently published a high prevalence (18%) of HBsAg in 19,408 HIV-infected patients from an African population [19]. In the current report, we present the largest study to date that explores the role of HBV and HIV co-infection on survival of HIV patients in an African setting. Knowledge of the prognostic effect of liver-related conditions, such as hepatitis B, which is highly endemic in the West African region, would help inform guidelines on screening and treatment with a potential to reduce excess mortality in HIV-infected patients.

Nigeria is categorised as a low income country, with a population of over 150 million and an estimated HIV prevalence of 4.4% [20] and HBsAg prevalence rates of up to 20% [21]. The aims of the present study were to assess the impact of hepatitis B infection, determined by HBsAg seropositivity, on the survival of HIV-infected individuals and to identify predictors of mortality during the HAART era in a large cohort of Nigerian patients.

## Methods

### Study setting and patients

The study was conducted as part of the “AIDS Prevention Initiative in Nigeria” (APIN) programme, affiliated to the Jos University Teaching Hospital (JUTH), Jos, Plateau State in the north-central region of Nigeria. Most patients enrolled into the JUTH/APIN programme were detected through Voluntary Counselling and Testing (VCT) services in adjacent communities. Hospitalised patients were investigated on clinical suspicion or referred from nearby health centres. Subjects found to be reactive to HIV by ELISA were confirmed by Western blot assay prior to enrolment. Liver function tests (LFTs), full blood count, HIV RNA and CD4 cell counts were measured in all recruited patients at baseline who were also screened for tuberculosis (TB), prior to commencement of HAART, following the Nigeria national HIV treatment guidelines (www.naca.gov.ng). From June 2004 to December 2010, 19,408 HIV-infected individuals had been recruited in the JUTH/APIN programme and were being monitored, on HAART and/or on anti-tuberculosis drugs. This number was estimated at 13% of all HIV-infected patients within the catchment area [20]. Onsite and offsite training was provided to personnel working at JUTH/APIN by HIV specialists from the Harvard School of Public Health (Boston, Massachusetts), Northwestern University (Chicago, Illinois) and Johns Hopkins University (Baltimore, Maryland), USA.

All patients were subjected to pre-treatment counselling. A patient tracking team, responsible for providing support to all enrolled patients (in terms of adherence fostering and to trace missing patients) was put in place in 2006. Treatment support groups (Hope Support), comprising mostly of HIV-infected patients, were set up within the clinic and surrounding communities.

PK, the guarantor of the study) obtained ethical approval for the present study from the Ethics Committee of JUTH and an additional Institutional Review Board (IRB) of Harvard School of Public Health, USA.

### Laboratory testing

**Figure 1:**
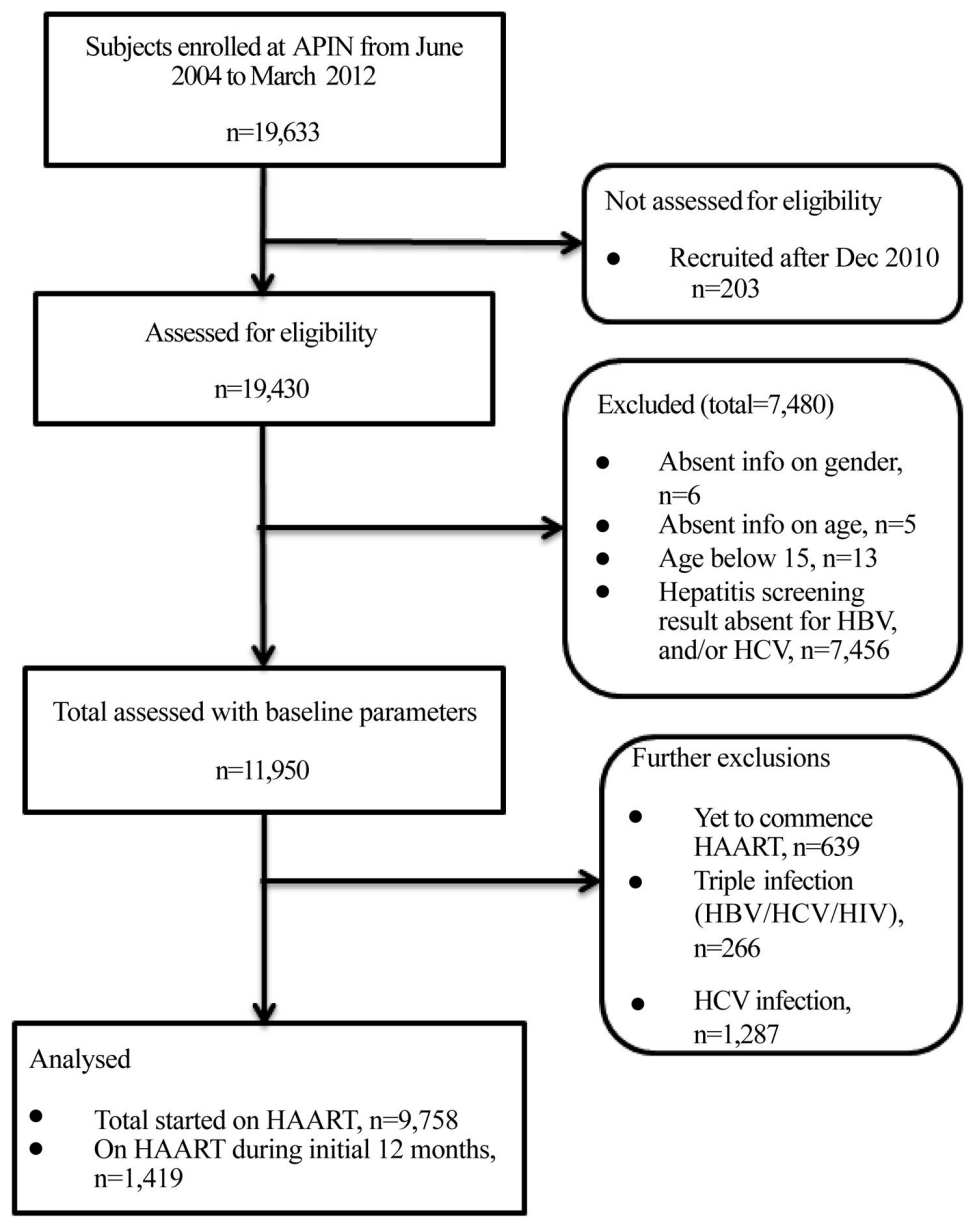
Profile of study cohort, AIDS Prevention Initiative in Nigeria, Jos University Teaching Hospital, Nigeria, 2004–2010.

Before recruitment into the APIN programme, subjects were screened for HIV using Rapid HIV test kits and subsequently confirmed by Western Blot assay. HBsAg was determined using Enzyme immunoassay (EIA) (Monolisa HBsAg Ultra3; BioRad, Hercules, CA, USA). HCV antibody was tested using third generation enzyme immunoassay (DIA.PRO Diagnostic, Bioprobessrl, Milan, Italy). HIV RNA levels were measured using Roche COBAS Amplicor HIV-1 monitor test version 1.5 (Roche Diagnostics, GmbH, Mannheim, Germany) with a detection limit of 400 copies/mL (Figure 1). Flow cytometry was used to determine CD4^+^ T-cell count (Partec, GmbH, Munster, Germany).

### Recruitment, treatment, monitoring and endpoints

Initial first-line antiretroviral (ARV) in 2004 included Stavudine/Zidovudine, Lamivudine and Efavirenz/Nevirapine. Abacavir and Truvada^®^ (Tenofovir plus Emtricitabine) have been administered since 2007 at APIN. Additionally, second-line treatment with protease inhibitors, in cases of treatment failure also became available since early 2007. However, during 2004 to 2006, Tenofovir and second-line agents were not available and HAART was not individualised. During 2007–2010, those patients commencing HAART that were co-infected with HBV, as well as those requiring second line agents, for virological failure were given ARVs containing Tenofovir.

Patients with CD4 cell counts below 200 cells/mm^3^were prescribed co-trimoxazole 960mg once a day, as prophylaxis for *P. neumocystisjiroveci* pneumonia (PCP). After an initial 4 weeks of fortnightly dispensing, HAART drugs were provided on a monthly basis to those patients on ART. Paper-based format of data entry was ensured by nurses, physicians and trained clinical officers and same day computerised data entry was carried out by Data Officers and supervised by a Data Manager. Personal information, medical history, physical examination, laboratory investigation and chest X ray reports comprised the initial records. Follow-up blood tests performed every 3 months, including LFTs, FBC, HIVRNA and CD4 cell count. Drug-related hepatotoxicity was defined as alanine aminotransferase (ALT) values ≥ 5 fold over the upper limit of the normal range (ULN) (41 iu/ mL for JUTH), or if ≥ 3.5 fold over ULN if baseline ALT was above ULN.

As HBV DNA and HCV RNA assays were not available routinely for this cohort, subjects were defined as having HBV and HCV infection if they tested positive for hepatitis B surface antigen (HBsAg) and hepatitis C antibody (HCV antibody), respectively on baseline blood samples.

The main end-point of our study was all-cause mortality. Most deaths were reported through the activities of the Tracking team and Hope Support groups. Other outcomes that were censored included those that stopped treatment (detected by pharmacy records), transfer to another health centre or lost to follow-up.

### Statistical analyses

The profile of how the patients were selected for analyses is presented in figure 1. Patients were included only if they had hepatitis B and hepatitis C screening performed on their sera at baseline. Apart from over 7000 patients who did not have one or both HBV and HCV screening, we additionally excluded those that were recruited after December 2010, and those with incomplete information (gender, age or under 15 yrs).

Kaplan-Meier models were used to estimate survival of the patient groups, while a Cox proportional hazard modelling was applied to identify independent predictors of mortality. Univariate analysis was embarked upon initially; and when a statistically significant association was detected, the variable was fitted into the multivariate model and hazard ratios calculated. Subsequently, we analysed the hazards of death for those patients on TDF-containing HAART, as well as for those not on TDF-containing HAART. Analyses were accomplished using MedCalc for Windows, version 9.5.0.0 (MedCalc Software, Mariakerke, Belgium). P values less than 0.05 were considered statistically significant.

## Results

### Baseline characteristics

Of the 9,748 adults that were commenced on HAART, 6,523 (66.9%) were women. The median age of the study population was 33 years (range 15–80 yrs.) and 5,915 (60.7%) of the patients had commenced HAART during the initial period, 2004–2006. The period of the study was defined based on availability of Tenofovir (HBV potent ARV) in the regimen for the patients commencing HAART. On this basis, initial period was 2004–2006 and later period was 2007–2010.

Summary of the baseline characteristics of patients recruited in the study is presented in Table 1. TB treatment was administered to 1,641 (16.8%) patients. For those that had CD4 results at baseline, there was a significantly higher median CD4 cell count in women than in men (M: 146 (95% CI=136–156) cells/mm^3^; F: 188 (95%CI=179–195) cells/mm^3^; p<0.0001). The median baseline HIV RNA load was log_10_4.52 (95% CI=4.50–4.55)(M:4.6(95%CI=4.6–4.7) copies/mL; F:4.5(95%CI=4.4–4.5) copies/mL; p<0.0001). HBsAg-positive rate was common in this study cohort; 1951 (20.0%). Liver disease was documented in only 23 (0.2%) of the patients.

### Survival analyses

**Figure 2:**
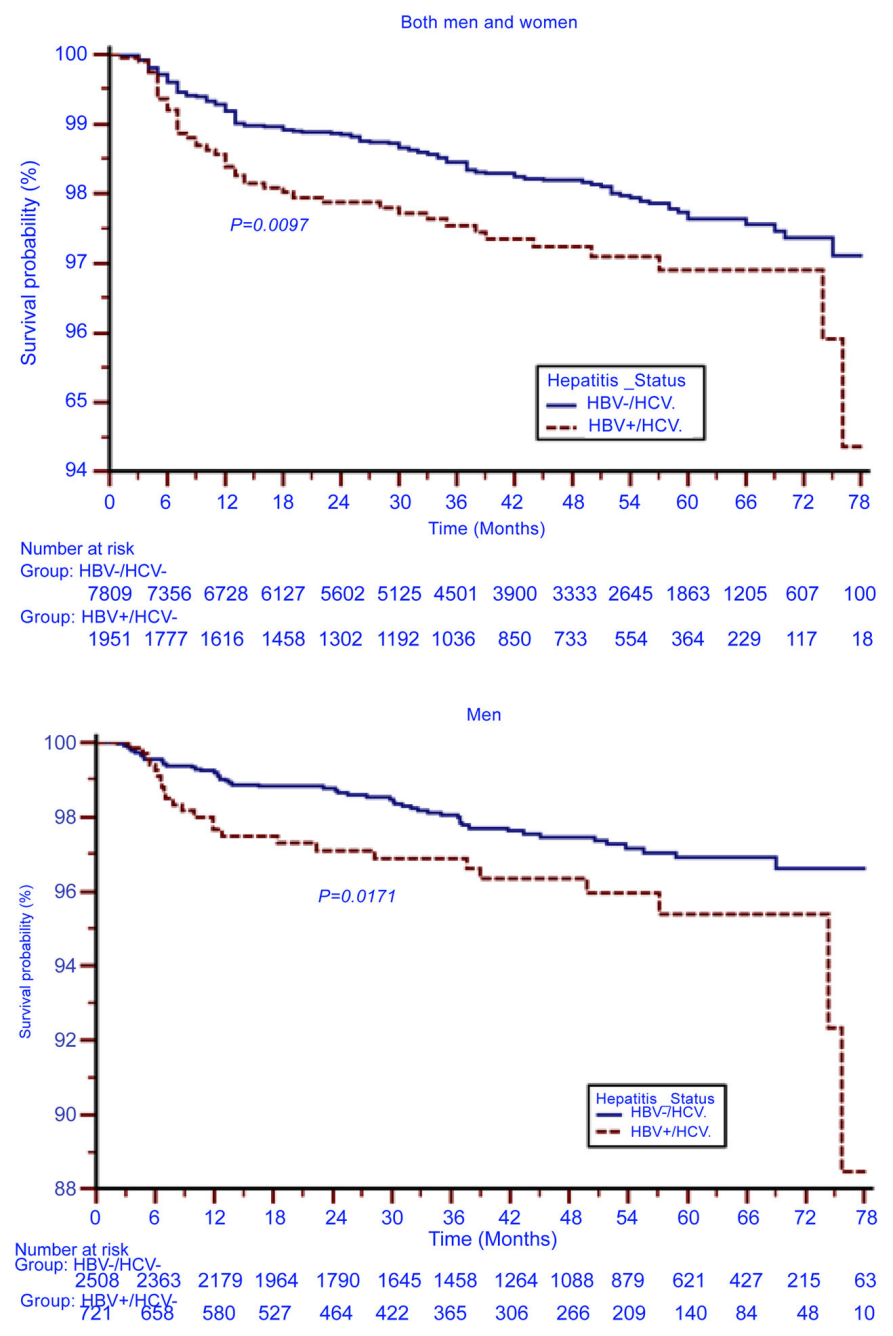
Kaplan-Meier survival curves according to hepatitis co-infection status for: both sexes; and men; APIN 2004–2010; HBV: Hepatitis B virus; HCV: hepatitis C virus.

Of those patients that were on HAART, 181 patients (1.9%) died during the follow-up period. One hundred and forty-three (79.0%) of those that died started ART during the initial period (2004–2006). Male gender, age ≥40 years at recruitment, co-morbid tuberculosis, HBsAg positivity, and recruitment during the initial phase of the study were all significantly associated with death (Table 2). Patients that were co-infected with HBV suffered a lower survival rate, compared to patient’s negative for HBsAg (Figure 2). The survival probability of HBsAg-positive versus HBsAg-negative patients at 78 months of follow-up was 94% and 97%, respectively (p=0.01). This was the case particularly for men; as HBV co-infection did not result in survival differences in women. HBsAg-positive men had lower survival, compared to HIV mono-infected men (HIV-positive/HBsAg-positive: 88.5%; HIV-positive/HBsAg-negative: 96.5%; p=0.04).

Fitting the non AIDS-related factors (ie excluding HIV viral load and CD4 cell count) into the Cox regression multivariate model, independent predictors of death were found to be male sex, co-morbid tuberculosis and HBV infection. The hazard of mortality was significantly reduced in patients who started HAART during 2007–2010 (HR: 0.64 [95% CI: 0.42–0.88]), compared to those enrolled during the earlier phase of the programme (p=0.0081). Intriguingly, HBsAg-positivity, male gender and enrolment that is more recent ceased to be significant in those that were on TDF-containing HAART.

As the majority of the deaths occurred during the initial follow-up period, we performed an additional Cox proportional hazard analysis of death during 12 months from recruitment into the study. Age ≥ 40 yrs at recruitment, HBV infection (HBsAg-positive) and enrolment during 2004–2006 were significant predictors of mortality during the early follow-up period.

## Discussion

In this African HIV-infected cohort of 9,758 patients on HAART, followed up for a median period of 41 months, we determined the impact of HBV infection on survival and studied independent predictors of mortality of HIV-infected patients undergoing antiretroviral therapy. HIV/HBV co-infection was found to be significantly associated with a lower probability of survival, compared to HIV mono-infection. On the other hand, recent recruitment into the APIN programme was significantly associated with reduced rate of death in both groups. Apart from HBV co-infection, tuberculosis and male sex were also significantly associated with mortality.

Two studies from South Africa found conflicting outcomes regarding the impact of HBV co-infection on HIV mortality [8,10]. Hoffman and colleagues in 2008 reported similar probability of deaths in HBV/HIV and HIV mono-infected patients over a 72-week period [10]. In contrast, Matthews and colleagues observed a significantly higher mortality in HBV co-infected patients, compared to HIV mono-infected 1,771 patients in South Africa over 48 weeks follow-up duration [8]. Our findings corroborated those of a large study of HIV-infected men in the US in which non AIDS-related mortality was highest among HIV/HBV-infected patients, despite being on HBV-active HAART [9] and in which no association between HBV infection and higher rates of HIV virologic failure was found. In contrast to the study in the US however, we found that HBV was not a predictor of mortality when these African HIV patients were on Tenofovir-containing HAART.

The reasons for the negative impact of HBsAg status on mortality are not fully understood, but it is unlikely to be related to accentuation of HIV replication, as we found similar levels of virologic suppression in co-infected versus HIV mono-infected patients in an earlier study of this cohort [19]. HBV infection in this population frequently occurs early in life and is postulated to precede HIV infection [22] by which time liver damage may be irreversible, annulling the anticipated response from HAART. Inflammatory response commonly occurs during the early phase of HAART [23] and could have contributed to the flaring of HBV infection [24].

We noted a higher mortality during the early phase of HAART in this study population. It is possible that this effect is due to late presentation of patients with more advanced disease. However, analysis restricted to only those who are taking Tenofovir suggests a beneficial impact in those with HBV.

Tuberculosis is a common co-morbidity of HIV-infected patients and was an independent predictor of mortality in the present study, in agreement with the results of other studies across Africa [25–27]. It is known that the relative risk of anti-tuberculous therapy-induced hepatotoxicity is higher in HBV/HIV co-infected patients than in HIV mono-infected individuals [28]. Symptomatic liver disease (particularly, jaundice) frequently prompts patients to seek alternative drugs, a common feature in African HIV populations [29,30], and/or which lead to treatment interruption and change to less potent regimens. If this had contributed in deaths in the present study cohort, such patients might have died at home, or away from the hospital affiliated to APIN and therefore may have be under represented in our data set.

Male gender was a significant predictor of mortality in the study cohort. Men had lower CD4 cell count at HAART commencement than women. Indeed, the CD4 cell count at commencement of HAART has been found to correlate with response and ultimately, survival [31]. Our data confirm this, as a significant trend of decreased death rate with increasing CD4 cell count prior to commencing HAART was found. Additionally, studies have reported that women generally do better than men to HIV treatment [32]. The prevention of mother to child transmission of HIV (PMTCT) services, which predated the onset of APIN, might have enabled more women to be used to HIV care and tolerating the side effects of ARVs than men.

Limitations encountered in our study include the following: First, the causes of death were not well ascertained as a result of suboptimal documentation. Precise dates of death of several patients were not recorded and we depended on information from carers, Hope Support and Tracking teams. We were thus limited in describing disease-specific deaths. Whenever any of the patients had any serious illness, such as liver disease, they were referred to specialist consultation in the affiliated hospital (JUTH). As no linkage of records exists currently between JUTH and APIN, considerable amounts of information that would have been relevant to our analyses may have been missed. Additionally, censoring subjects who discontinued treatment, transferred to other centres of care and or lost to follow up may have introduced bias to the obtained results.

Second, confirmation of HBV viraemic status by molecular methods was not carried out in this population as this current study was designed to utilise already obtained data and molecular diagnosis of hepatitis is not routine in the study population (we had shown, in an unpublished data that HBV viraemia was positive in about 98% of HBsAg-positive subpopulation of this study cohort). It would be of interest to appropriately categorise the patients by their viraemic status, which would indeed enhance the power of this study.

In conclusion, HBV infection, tuberculosis and male gender were significantly associated with mortality, following commencement of HAART therapy. Patients recruited during the period, coinciding with increased access to Tenofovir-based treatment experienced less deaths. Indeed, HBV was not a significant predictor of mortality in those patients that were being administered Tenofovir-containing HAART. Long-term follow-up and enhanced monitoring are needed to assess incidence of end-stage liver disease, hepatocellular carcinoma and disease-specific deaths in this cohort. These data support an active approach to identifying all HIV/HBV co-infected individuals in resource poor settings, if Tenofovir is not routinely offered as first line treatment [33].

## Figures and Tables

**Table 1:** Baseline characteristics and associated deaths among HIV-infected patients in Nigeria, 2004–2010.

Characteristic	Total n=9,758	Deaths n (%)	p value	Male n=3,229	Deaths n (%)	Female n=6,530	Deaths n %)
Age-group (years)			0.008*				
15–29	3,147	45(1.4)		393	4(4.0)	2,754	41(1.5)
30–39	3,967	73(1.8)		1,378	31(2.2)	2,589	42(1.6)
40–49	2,001	47(2.3)		1,069	35(3.3)	932	12(1.3)
50+	644	16(2.5)		389	9(2.3)	255	7(2.7)
HBsAg status			0.034				
HBsAg +ve	1,950	48(2.5)		721	25(3.5)	1,230	23(1.9)
HBsAg –ve	7,808	133(1.7)		2,508	54(2.2)	5,300	79(1.5)
Liver disease			0.9				
Present	23	0(0.0)		5	0(0.0)	18	0(0.0)
Absent	9,728	181(1.9)		3,220	79(2.5)	6,508	102(1.9)
TB diagnosis			<0.0001				
Present	1,640	55(3.4)		743	30(4.0)	898	25(2.8)
Absent	8,118	126(1.6)		2,486	49(2.0)	5,632	77(1.4)
HIV RNA at baseline (viral copies/mL)			0.06*				
<400	1,267	25(2.0)		408	11(2.7)	859	14(1.6)
400–9,999	1,950	23(1.2)		565	9(1.6)	1,385	14(1.0)
10,000–29,999	1,523	22(1.4)		427	10(2.3)	1,096	12(1.1)
≥30,000	5,019	111(2.2)		1,829	49(2.7)	3,190	62(1.9)
CD4 cell count at baseline (cells/mL)			0.003*				
<200	1,599	24(1.5)		605	7(1.2)	994	17(1.7)
200–499	1,050	4(0.4)		295	1(0.3)	755	3(0.4)
≥500	136	0(0.0)		24	0(0.0)	112	0(0.0)
Period of enrolment			<0.0001				
2004–2006	5,920	143(2.4)		1,963	67(3.4)	3,958	76(1.9)
2007–2010	3,838	38(1.0)		1,266	12(0.9)	2,572	26(1.0)

* p values indicate comparison for trend

**Table 2:** Multivariate analyses of predictors of mortality in HIV-infected individuals, AIDS Prevention Initiative in Nigeria JUTH, 2004–2010.

HAART	Covariate	HR (95% CI)	SE	Coefficient	p value
All time	Male	1.5(1.08–2.00)	0.15	0.4	0.0134
	HBsAg +ve	1.5(1.09–2.11)	0.17	0.4	0.0129
	Later recruitment (2007–2010)	0.6(0.42–0.88)	0.19	−0.5	0.0081
	TB diagnosis	2.2(1.57–2.96)	0.16	0.8	<0.0001
Initial 12 months					
	HBsAg +ve	2.1(1.32–3.21)	0.23	0.7	0.0015
	40–49 years	1.9(1.16–3.07)	0.25	0.6	0.011
	≥50	2.5(1.31–4.80)	0.33	0.9	0.0057
	Earlier recruitment (2004–2006)	6.3(3.86–10.31)	0.25	1.8	<0.0001
None-TDF-based HAART	Male	1.8(1.16–2.68)	0.21	0.6	0.0084
	HBsAg +ve	4.3(2.60–6.95)	0.25	1.4	<0.0001
	Latter recruitment (2007–2010)	0.3(0.14–0.57)	0.36	−1.2	0.0004
TDF-based HAART	TB diagnosis	2.9(1.94–4.39)	0.21	1.1	<0.0001

TDF = Tenofovir

## Abbreviations

HAART: Highly Active Antiretroviral Therapy
HBV: Hepatitis B Virus
HIV: Human Immunodeficicency Virus
CI: Confidence Interval
ART: Antiretroviral Therapy
AIDS: Acquired Immunodeficiency Syndrome
APIN: AIDS Prevention Initiative in Nigeria
JUTH: Jos University Teaching Hospital
VCT: Voluntary Counselling and Testing
ELISA: Enzyme-Linked Immunosorbent Assay
EIA: Enzyme Immunoassay
LFT: Liver Function Test
RNA: Ribonucleic Acid
IRB: Institutional Review Board
HCV: Hepatitis C Virus
USA: United States of America
PMTCT: Prevention of Mother to Child Transmission
Arvs: Antiretrovirals
